# Amniotic Fluid Protein Profiles of Intraamniotic Inflammatory Response to *Ureaplasma* spp. and Other Bacteria

**DOI:** 10.1371/journal.pone.0060399

**Published:** 2013-03-26

**Authors:** Marian Kacerovsky, Peter Celec, Barbora Vlkova, Kristin Skogstrand, David M. Hougaard, Teresa Cobo, Bo Jacobsson

**Affiliations:** 1 Biomedical Research Center, University Hospital Hradec Kralove, Hradec Kralove, Czech Republic; 2 Department of Obstetrics and Gynecology, Charles University in Prague, Faculty of Medicine Hradec Kralove, Hradec Kralove, Czech Republic; 3 Institute of Molecular Biomedicine, Comenius University in Bratislava, Bratislava, Slovak Republic; 4 Department of Clinical Biochemistry and Immunology, Statens Serum Institut, Copenhagen, Denmark; 5 Maternal Fetal Medicine Department, Hospital Clinic, IDIBAPS, Barcelona, Spain; 6 Department of Obstetrics and Gynecology, Sahlgrenska University Hospital, Gothenburg, Sweden; 7 Institute of Public Health, Oslo, Norway; Institut Jacques Monod - UMR 7592 CNRS - Université Paris Diderot, France

## Abstract

**Objective:**

This study aimed to evaluate the amniotic fluid protein profiles and the intensity of intraamniotic inflammatory response to *Ureaplasma* spp. and other bacteria, using the multiplex xMAP technology.

**Methods:**

A retrospective cohort study was undertaken in the Department of Obstetrics and Gynecology, University Hospital Hradec Kralove, Czech Republic. A total of 145 pregnant women with preterm prelabor rupture of membranes between gestational age 24+0 and 36+6 weeks were included in the study. Amniocenteses were performed. The presence of *Ureaplasma* spp. and other bacteria was evaluated using 16S rRNA gene sequencing. The levels of specific proteins were determined using multiplex xMAP technology.

**Results:**

The presence of *Ureaplasma* spp. and other bacteria in the amniotic fluid was associated with increased levels of interleukin (IL)-6, IL-8, IL-10, brain-derived neurotropic factor, granulocyte macrophage colony stimulating factor, monocyte chemotactic protein-1, macrophage inflammatory protein-1, and matrix metalloproteinasis-9. *Ureaplasma* spp. were also associated with increased levels of neurotropin-3 and triggering receptor expressed on myeloid cells-1.

**Conclusions:**

The presence of *Ureaplasma* spp. in the amniotic fluid is associated with a slightly different protein profile of inflammatory response, but the intensity of inflammatory response to *Ureaplasma* spp. is comparable with the inflammatory response to other bacteria.

## Introduction

Microbial invasion of the amniotic cavity (MIAC) is often found in women with preterm prelabor rupture of membranes (PPROM). MIAC is detected in approximately 20% to 50% of these patients, depending on the testing method [1], [2]. Although cultivation of amniotic fluid is still considered the gold standard for the identification of MIAC, the use of molecular techniques enable the detection of uncultivated or difficult-to-cultivate bacteria as well.

A variety of microorganisms have been isolated from the amniotic fluid in pregnancies complicated by both PPROM and preterm labor, *Ureaplasma* spp. being the most prevalent [1], [2], [3]. *Ureaplasma* spp. account for up to 60% of all detected bacteria in the amniotic fluid of women with PPROM, depending on the use of cultivation-based or the more sensitive non-cultivation-based techniques [2]. *Ureaplasma* spp. can colonize the choriodecidual space without eliciting an inflammatory response due to their low pathogenicity and the immunosuppressive properties of the choriodecidua [4]. On the other hand, when *Ureaplasma* spp. appear inside the amniotic cavity, they can induce a strong intraamniotic inflammatory response with the development of histological chorioamnionitis and even signs of a maternal inflammatory response [2], [5]. In addition, current studies have shown that *Ureaplasma* spp. have a capacity similar to that of other bacteria to induce both intraamniotic and maternal inflammatory responses [4], [5].

Protein profiling of amniotic and cervical fluid has been used successfully to assess and predict MIAC in pregnancies complicated by PPROM and preterm labor with intact membranes [6], [7], [8]. In addition, recent *ex vivo* explant model studies have suggested that cytokine and protein response to bacteria seems to be pathogen dependent [9], [10], [11], [12].

Therefore, the main aim of this study was to evaluate the diversity between the amniotic fluid protein profiles of intraamniotic inflammatory response to *Ureaplasma* spp. and those concerning other bacteria by employing multiplex xMAP technology. A second aim was to compare the intensity of intraamniotic inflammatory responses evoked by *Ureaplasma* spp. with that produced by other bacteria.

## Materials and Methods

### Sample collection

Between July 2008 and October 2010, we conducted a prospective cohort study of women with PPROM between 24+0 and 36+6 weeks' gestation, who were admitted to the Department of Obstetrics and Gynecology, University Hospital Hradec Kralove, Czech Republic. Women of maternal age ≥18 years and with a singleton pregnancy were eligible for enrollment in the study. The exclusion criteria were as follows: presence of maternal complications (i.e., hypertension, preeclampsia, diabetes mellitus, and thyroid disease), ultrasound signs of fetal growth restriction, vaginal bleeding, signs of fetal hypoxia, and structural malformations or chromosomal abnormalities of the fetus. Gestational age was established using the first trimester ultrasound evaluation for all pregnancies.

PPROM was defined as the leakage of amniotic fluid prior to the onset of labor (by at least two hours). This condition was diagnosed using a sterile speculum examination, which confirmed the pooling of amniotic fluid in the vagina, in association with a positive test for the presence of insulin-like growth factor binding protein (ACTIM PROM test; Medix Biochemica, Kauniainen, Finland) in the vaginal fluid. Ultrasound-guided transabdominal amniocentesis was performed on admission prior to the administration of corticosteroids, antibiotics, or tocolytics, and approximately 5 mL of amniotic fluid were aspirated and divided into two tubes.

The study was approved by the University Hospital Hradec Kralove review board committee (March 19, 2008; No. 200804 SO1P), and written informed consent was obtained from all participants.

### Amniotic fluid analyses

First tubes of uncentrifuged amniotic fluid were transported to the laboratory for DNA isolation and 16S rRNA gene sequencing. Protease inhibitors (Complete^TM^ Mini, EDTA-free Protease Inhibitor Cocktail; Roche Diagnostics, Basel, Switzerland) were added (40 µL per 1 mL of amniotic fluid) to the second tubes, which were centrifuged for 15 minutes at 300×*g* to remove cells and debris, filtered (0.22 µm) using a Syringe-driven filter (TPP, Trasadingen, Switzerland), divided into aliquots, and stored at −70°C until analysis.

The levels of interleukin (IL)-1β, IL-6, IL-8, IL-10, IL-12, IL-17, IL-18, soluble IL-6 receptor α, adiponectin, brain-derived neurotropic factor (BDNF), C-reactive protein (CRP), granulocyte macrophage colony stimulating factor (GM-CSF), insulin-like growth factor-binding protein (IGFBP)-1, IGFBP-3, interferon-γ, leptin, monocyte chemotactic protein-1 (MCP-1), migration inhibiting factor (MIF), macrophage inflammatory protein-1α (MIP-1α), matrix metalloproteinasis-9 (MMP-9), neutropin-3 (NT-3), regulated on activation normal T-expressed and secreted (RANTES), tumor necrosis factor (TNF)-α, TNF-β, soluble tumor necrosis factor receptor-1 (sTNF-R1), and triggering receptor expressed on myeloid cells-1 (TREM-1) in the amniotic fluid were analyzed using a multiple sandwich immunoassay based on flowmetric Luminex xMAP technology, in accordance with previously published papers[13], [14], [15]. Only proteins with detectable amniotic fluid levels in more than 50% of the samples were included in the analyses. The following eight proteins were excluded from further analyses because of low levels: IL-1β detected in 17%, IL-12 in 9%, IL-17 in 8%, IL-18 in 13%, interferon-γ in 0%, RANTES in 17%, TNF-α in 10%, and TNF-β in 11%.

### Detection of bacteria in amniotic fluid

DNA was extracted from the samples using a QIAmp DNA Mini kit according to the instructions of the manufacturer (Qiagen, Hilden, Germany). The isolated DNA was used as a template for polymerase chain reaction (PCR) of the 16S rRNA gene with primers at conserved sites flanking a variable sequence used for terminal fragment length restriction polymorphism analysis: 27F (5′-AGAGTTTGATCCTGGCTCAG-3′) and 1492R (5′-GGTTACCTTGTTACGACTT-3′). This assay is widely used in microbiological research to study complex samples as part of the terminal restriction fragment length polymorphism (tRFLP). The primers as well as the assay are routinely used in a number of applications including the characterization of microflora of the nasal and oral cavity, gastric microflora, and the microflora of the gut [16], [17], [18], [19], [20]. The method has been extensively optimized on bacterial cultures and saliva [21], [22], [23]. The resulting protocol was used in this study. The endpoint PCR analysis was performed using the PCR instrument Eppendorf Mastercycler ep (Eppendorf AG, Hamburg, Germany). The amplification primers were designed to detect fragment of the prokaryotic 16S rRNA gene. Sequences of the used primers were described previously [22]. The reactions were set up in a total volume of 20 µL, containing 10×Taq Buffer, dNTP Mix (0.2 mM of each), 1.0 µM of each primer, 1.5 mM of MgCl_2_, 1.25 U of Taq DNA Polymerase (Thermo Scientific), and 1.5 µL of the template. Cycling conditions were 95°C for 15 min, followed by 35 cycles of three-step cycling of 94°C for 30 sec, 50°C for 30 sec, 72°C for 120 sec; with final polymerization step 12 min at 72°C). PCR products were checked using agarose gel electrophoresis, purified by ethanol precipitation, and sequenced on an ABI 3100 genetic analyzer (Life Technologies, Carlsbad, USA). Chromas 2.33 (Technelysium Pty Ltd, South Brisbane, Australia) and the online basic local alignment search tool (BLAST) algorithm were used for sequence analysis [24]. Based on the sequencing run either the resulting sequence was used for a BLAST search or if the run was unreadable with superimposed sequences read in parallel, an infection with multiple species was diagnosed.

### Statistical analysis

Demographic and clinical characteristics were compared using the unpaired *t*-test (presented as the mean±SEM) and the nonparametric Mann-Whitney *U* test, and are presented as median (range). Categorical variables were compared using Fischer's exact test and are presented as number (%). The normality of the data was tested using the D'Agostino and Pearson omnibus normality test. Differences were considered statistically significant at *p*<0.05 using a two-sided alternative hypothesis. Statistical analyses were performed using SPSS 19.0 for Windows XP OS (SPSS Inc., Chicago, IL, USA).

## Results

### Demographical and clinical characteristic

A total of 145 women with PPROM were recruited during the study period. Of these women, only 106 (73%) were included in the analyses, because aliquots of amniotic fluid samples were not available for 39 (20%) women. Of the 106 women, 25 (24%) presented with positive 16S rRNA in the amniotic fluid. Sequencing revealed *Ureaplasma* spp. in 12% of the women (13/106). Other bacteria were detected in 12% (12/106). Polymicrobial finding (*Sneathia sanguinegens* and *Leptotrichia amnionii*) was detected in the amniotic fluid of one woman. Table 1 presents the microorganisms identified in the amniotic fluid from women included in the analyses. Table 2 shows the demographic and clinical characteristics of women with *Ureaplasma* spp., of women with other bacteria, and of women without any bacteria in the amniotic fluid. Women with either *Ureaplasma* spp. or other bacteria had lower birth weight of newborn, and a higher rate of histological chorioamnionitis and funisitis. In addition, women with other bacteria in the amniotic fluid had lower gestational age at sampling and delivery. No differences were found between the groups with *Ureaplasma* spp. and with other bacteria. All women in the study were Caucasian, as defined by self-report.

**Table 1:** The bacteria found in the amniotic fluid using 16S rRNA gene sequencing.

The microorganisms	Number of cases
*Ureaplasma* spp.	13
*Granulicatella elegans*	1
*Haemophilus influenzae*	2
*Lactobacillus crispatus*	1
*Lactobacillus gallinarum*	1
*Leptotrichia amnionii*	2
*Peptostreptococcus anaerobicus*	1
*Sneathia sanguinegens*	1
*Streptococcus agalactiae*	2
*Streptococcus pneumoniae*	1
*Staphylococcus* spp.	1

The variables are presented as number.

Two microorganisms in the amniotic fluid were detected in one woman (*Sneathia sanguinegens*+*Leptotrichia amnionii*).

**Table 2:** Maternal and newborn characteristics based on the presence of *Ureaplasma* spp. and other bacteria, and absence of the bacteria in the amniotic fluid.

	The presence of *Ureaplasma* spp. (n = 13)	The presence of other bacteria (n = 12)	The absence of the bacteria (n = 81)	*p-*value^1^	*p-*value^2^	*p-*value^3^
Maternal age (years)	27.5±1.8	30.6±1.7	30.8±0.7	0.09	0.80	0.22
Nulliparous	6 (46%)	4 (33%)	48 (59%)	0.39	0.12	0.69
Previous spontaneous abortion	1 (8%)	2 (17%)	15 (19%)	0.27	0.72	0.59
Prepregnancy body mass index	19.4 (17.3–35.7)	22.4 (18.5–29.0)	21.7 (16.3–40.6)	0.07	0.52	0.45
Smoking during pregnancy	3 (23%)	2 (17%)	12 (15%)	0.43	1.00	1.00
PPROM to AMC interval (hours)	5 (1–22)	4 (2–23)	6 (3–18)	0.95	0.20	0.35
Gestational age at AMC	32+0 (25+0–36+3)	30+3 (24+0–35+4)	33+5 (24+0–36+5)	0.13	**0.01**	0.24
Gestational age at delivery	32+1 (25+4–36+4)	30+6 (24+2–35+5)	33+5 (24+0–36+5)	0.11	**0.009**	0.20
CRP level at admission (mg/L)	11.0 (1.0–25.0)	10.0 (0–59.0)	5.8 (0–82.0)	0.08	0.13	0.75
WBC count at admission (x10^9^/L)	12.9 (7.0–27.0)	14.0 (4.0–19.0)	12.0 (7.0–24.0)	0.25	0.61	0.98
Vaginal delivery	10 (77%)	6 (50%)	61 (75%)	1.00	0.09	0.22
Cesarean section	3 (23%)	6 (50%)	20 (25%)	1.00	0.09	0.22
Birth weight (grams)	1629±144	1515±218	2103±73	**0.006**	**0.001**	0.51
PPROM to delivery interval (hours)	51 (8–244)	51 (8–120)	28 (5–217)	0.43	0.14	0.70
Apgar score in 5 minutes	9 (0–10)	9 (0–10)	9 (1–10)	0.57	**0.02**	0.09
Apgar score in 10 minutes	10 (6–10)	9 (7–10)	10 (6–10)	0.92	**0.002**	0.08
The presence of HCA	11 (85%)	11 (92%)	36 (44%)	**0.01**	**0.004**	1.00
The presence of funisitis	5 (38%)	5 (58%)	6 (7%)	**0.007**	**0.005**	1.00

Abbreviations: PPROM-preterm prelabor rupture of membranes; AMC-amniocentesis; CRP–C-reactive protein; WBC–white blood cells; HCA–histological chorioamnionitis.

*p*-value^1^ the comparison between groups with the presence of *Ureaplasma* spp. and without bacteria in amniotic fluid.

*p*-value^2^ the comparison between groups with the presence of other bacteria and without bacteria in amniotic fluid.

*p*-value^3^ the comparison between groups with the presence of *Ureaplasma* spp. and other bacteria in amniotic fluid.

Continuous variables were compared using parametric t-test (presented as mean ± SEM) or a nonparametric Mann-Whitney *U* test [presented as median (range)]. Categorical variables were compared using Fisher exact test and presented as number (%).

Statistically significant differences are marked in bold.

### Amniotic fluid protein profile of women with *Ureaplasma* spp

The presence of *Ureaplasma* spp. in the amniotic fluid was associated with changes in 10 of 18 measured amniotic fluid proteins. Amniotic fluid levels of IL-6, IL-8, IL-10, BDNF, GM-CSF, MCP-1, MIP-1α, MMP-9, NT-3, and TREM-1 were higher in women with *Ureaplasma* spp. in the amniotic fluid compared with women with no bacteria in the amniotic fluid (see Table 3). The results are not adjusted for gestational age at sampling because no difference was found.

**Table 3:** Amniotic fluid levels of selected proteins in women with the presence of *Ureaplasma* spp. and without the bacteria in the amniotic fluid.

	The presence of *Ureaplasma* spp. (n = 13)		Without bacteria (n = 81)		*p-*value
	Median (range)	Mean±SEM	Median (range)	Mean±SEM	
IL-6	1567 (61–5090)	2193±463	337 (28–4232)	639±99	**<0.0001**
IL-8	456 (4–4000)	936±385	58 (4–4000)	260±76	**0.002**
IL-10	183 (4–2199)	469±190	15 (4–106)	18±2	**<0.0001**
sIL-6rα	523 (65–2987)	887±262	225 (38–1843)	362±42	0.09
Adiponectin	56 (4–357)	116±31	40 (12–377)	58±6	0.14
BDNF	269 (35–930)	308±67	118 (10–481)	137±12	**0.002**
CRP	3357 (1278–17786)	4651±1214	2037 (223–24601)	3511±456	0.06
GM-CSF	38 (4–97)	39±7	13 (4–99)	19±2	**0.002**
IGFBP-1	33 (12–173)	48±14	24 (5–185)	40±4	0.79
IGFBP-3	30 (22–39)	32±2	31 (6–43)	29±10	0.49
Leptin	818 (234–4564)	1210±332	930 (42–7672)	1219±150	0.81
MCP-1	846 (38–2500)	1023±261	142 (37–2500)	324±49	**0.004**
MIF	7 (2–50)	11±3	5 (13–50)	8±1	0.55
MIP-1α	718 (96–2567)	1059±264	48 (4–377)	65±7	**<0.0001**
MMP-9	4193 (956–68255)	14966±5425	2021 (461–16325)	3086±311	**0.004**
NT-3	551 (217–957)	571±71	368 (120–1259)	423±22	**0.04**
sTNF-R1	1090 (249–4717)	1450±388	369 (135–3943)	830±108	0.05
TREM-1	354 (114–2790)	520±195	159 (50–680)	183±13	**0.001**

Continuous variables were compared using a nonparametric Mann-Whitney *U* test [presented as median (range) and mean±SEM].

Adiponectin, IGFB-1, IGFB-3, and MIF are presented in ng/mL; other mediators are showed in pg/mL.

Statistically significant differences are marked in bold.

### Amniotic fluid protein profile of women with other bacteria

The presence of other bacteria in the amniotic fluid was related to changes in the amniotic fluid levels of five and seven proteins in crude analysis and adjusted analysis for gestational age at sampling, respectively (see Table 4). The amniotic fluid levels of IL-6, IL-8, IL-10, GM-CSF, MCP-1, MIP-1α, and MMP-9 were higher in women with other bacteria compared with those without bacteria in the amniotic fluid after adjustment for gestational age at sampling.

**Table 4:** Amniotic fluid levels of selected proteins in women with other bacteria and without bacteria in the amniotic fluid.

	The presence of other bacteria (n = 12)		Without the bacteria (n = 81)		*p-*value	*p*-value^*^
	Median (range)	Mean±SEM	Median (range)	Mean±SEM		
IL-6	1413 (17–6659)	2370±616	337 (28–4232)	639±99	**0.001**	**<0.0001**
IL-8	469 (12–1011)	481±108	58 (4–4000)	260±76	**0.001**	**0001**
IL-10	75 (12–1692)	374±173	15 (4–106)	18±2	**0.001**	**0.001**
sIL-6rα	660 (90–564)	688±160	225 (38–1843)	362±42	0.10	0.37
Adiponectin	67 (24–178)	77±14	40 (12–377)	58±6	0.13	0.72
BDNF	206 (10–866)	280±78	118 (10–481)	137±12	0.07	0.13
CRP	1710 (162–15417)	4394±1355	2037 (223–24601)	3511±456	0.79	0.58
GM-CSF	24 (4–161)	50±15	13 (4–99)	19±2	0.08	**0.002**
IGFBP-1	26 (12–787)	31±6	24 (5–185)	40±4	0.27	0.08
IGFBP-3	35 (20–44)	33±2	31 (6–43)	29±10	0.17	0.83
Leptin	1168 (138–6226)	1617±476	930 (42–7672)	1219±150	0.53	0.52
MCP-1	657 (61–2500)	922±221	142 (37–2500)	324±49	**0.003**	**0.005**
MIF	10 (2–33)	11±3	5 (13–50)	8±1	0.29	0.80
MIP-1α	566 (32–6355)	1946±735	48 (4–377)	65±7	**<0.0001**	**<0.0001**
MMP-9	4751 (820–87534)	14228±7046	2021 (461–16325)	3086±311	0.08	**0.008**
NT-3	363 (235–2368)	692±194	368 (120–1259)	433±22	0.45	0.28
sTNF-R1	995 (196–7517)	2110±733	369 (135–3943)	830±108	0.09	0.21
TREM-1	175 (43–2248)	490±207	159 (50–680)	183±13	0.35	0.05

Continuous variables were compared using a nonparametric Mann-Whitney *U* test [presented as median (range) and mean±SEM].

*p*-value the comparison between groups with the presence of other bacteria and without bacteria in amniotic fluid.

*p*-value* Spearman partial correlation was performed to adjust by gestational age at sampling.

Adiponectin, IGFB-1, IGFB-3, and MIF are presented in ng/mL; other mediators are showed in pg/mL.

Statistically significant differences are marked in bold.

### Intensity between intraamniotic and maternal inflammatory response evoked by *Ureaplasma* spp. and other bacteria

Only those proteins that showed changes in the presence of either *Ureaplasma* spp. or other bacteria in the amniotic fluid (IL-6, IL-8, IL-10, BDNF, GM-CSF, MCP-1, MIP-1α, MMP-9, NT-3, and TREM-1) were included in the analysis. No differences in their levels were observed between women with *Ureaplasma* spp. and those with other bacteria (see Table 5). Furthermore, we did not find any difference between maternal serum CRP level and white blood cell count (see Table 2).

**Table 5:** Amniotic fluid levels of proteins involved in intraamniotic inflammatory responses to *Ureaplasma* spp. and other bacteria.

	The presence of *Ureaplasma* spp. (n = 13)		The presence of other bacteria (n = 12)		*p*-value
	Median (range)	Mean±SEM	Median (range)	Mean±SEM	
IL-6	1567 (61–5090)	2193±463	1413 (17–6659)	2371±616	1.00
IL-8	456 (4–4000)	936±349	469 (12–1011)	482±108	0.98
IL-10	183 (4–2199)	469±190	75 (12–1692)	18±2	0.66
sIL-6rα	523 (65–2987)	887±262	660 (90–564)	688±160	1.00
BDNF	269 (35–930)	308±67	206 (10–866)	280±78	0.45
GM-CSF	38 (4–97)	39±7	24 (4–161)	50±15	0.94
MCP-1	846 (38–2500)	1023±261	657 (61–2500)	922±221	0.98
MIP-1α	718 (96–2567)	1059±264	566 (32–6355)	1946±735	0.83
MMP-9	4193 (956–68255)	14966±5425	4751 (820–87534)	14228±7046	0.62
NT-3	551 (217–957)	571±71	363 (235–2368)	692±194	0.45
TREM-1	354 (114–2790)	520±195	175 (43–2248)	490±207	0.21

Continuous variables were compared using a nonparametric Mann-Whitney *U* test [presented as median (range) and mean±SEM].

*p*-value the comparison between groups with the presence of *Ureaplasma* spp. and other bacteria in amniotic fluid.

Levels of mediators are showed in pg/mL.

## Discussion

A recent study showed that different types of bacteria evoke varied inflammatory responses in an explant model with fetal membranes [9]. Therefore, we hypothesized that the inflammatory response to different types of bacteria could be associated with diverse protein profiles that play a role in this process. The diversity of bacteria found in the amniotic fluid from pregnancies complicated by PPROM prevented us from creating more than two main cohorts of women with bacteria in their amniotic fluid. These two main cohorts consisted of women with *Ureaplasma* spp. and women with bacteria other than *Ureaplasma* spp.

The following are the key findings from this study: i) the presence of *Ureaplasma* spp. in the amniotic fluid is associated with a slightly different, more extensive protein profile of inflammatory response; ii) the changes in amniotic fluid IL-6, IL-8, IL-10, GM-CSF, MCP-1, MIP-1α, and MMP-9 are common for both *Ureaplasma* spp. and other bacteria; and iii) the intensity of inflammatory response to *Ureaplasma* spp. is fully comparable with that to bacteria other than *Ureaplasma* spp.

Different types of bacteria have been found to produce varied inflammatory responses in an explant model with fetal membranes [9], [10], [11], [12]. Menon et al. found an association between the stimulation of fetal membranes by *Ureaplasma parvum* and an increase in TNF-α and IL-10, but not in IL-1β, IL-6, IL-8, and interferon-γ levels in tissue explant model. On the other hand, stimulations by other bacteria, such as *Escherichia coli*, *Streptococcus agalactiae*, and *Gardanerella vaginalis*, were related to higher levels of IL-1β, IL-6, and IL-10[9]. With this knowledge, we hypothesized that the presence of bacteria with a higher virulence potential in the amniotic fluid would be associated with a more pronounced and extensive protein profile of intraamniotic inflammatory response.

Innate immunity provides prompt protection against the threat of invading bacteria and other microorganisms. Specific motifs on bacterial surfaces are recognized by pattern-recognition receptors, and the activation of these receptors leads to the release of cytokines, chemokines, and other inflammatory mediators. Our results demonstrate that the protein profiles of intraamniotic inflammatory response to *Ureaplasma* spp. and to other bacteria are very similar, involving cytokines (IL-6, IL-10, and GM-CSF), chemokines [IL-8 (CXCL8 chemokine), MCP-1 (CCL2 chemokine), and MIP-1α (CCL3 chemokine)], and proteins involved in the breakdown of the extracellular matrix (MMP-9). A solid body of evidence indicates that IL-6, IL-8, and IL-10, along with IL-1β and TNF-α (unfortunately, the levels of the latter two were below the detection limit in more than 50% of the cases), play a central role in the mechanism of intraamniotic inflammation [25], [26]. GM-CSF is a cytokine with the ability to generate monocytes, neutrophils, and other granulocytes from precursor cells. Therefore, its higher levels could be one explanation for the observed local accumulation of these cells at sites of inflammation [27]. We found chemokines to be responsible for recruitment of first-line (IL-8, MIP-1α) and second-line (MCP-1) defense cells. Neutrophils that belong to the first line of the defense mechanism against invading microorganisms are thought to play an important role in the secretion of MMP-9, which is released following stimulation by IL-8 and other inflammatory mediators [28], [29]. Moreover, MMP-9 plays an important role in inflammation due to the degradation of matrix protein and further activation of cytokines and chemokines [30]. Two specific proteins, NT-3 and TREM-1, were involved only in the intraamniotic inflammatory response to *Ureaplasma* spp. in the amniotic fluid. Their exact roles in this pathway remain unclear.

In a study by Oh et al., it was proposed that genital mycoplasmas (*Ureaplasma* spp. and *Mycoplasma hominis*) exhibit more intense intraamniotic and maternal inflammatory responses than other microorganisms. We did not find any difference between the intensity of intraamniotic inflammatory response, as determined by the levels of 10 specific proteins, to *Ureaplasma* spp. and other bacteria. Moreover, no difference was revealed in maternal inflammatory response as well. We assume that the employment of different approaches for the detection of bacteria (cultivation vs. non-cultivation) may explain this discrepancy, making comparison of the results difficult.

In our study, MIAC was revealed in 24% women with PPROM. This rate is lower compare to data presented in the recent study by DiGiulio et al., where bacteria were identified in 45% of PPROM pregnancies [31], [32]. Nevertheless, the results cannot be directly compared between their and our study. DiGiulio et al. included women with PPROM with gestational age between 15+0−36+6 weeks and they also included women with different ethnic background [31]. In our study, we only included Caucasian women with gestational age between 24+0−36+6 weeks. Lower gestational age is associated with higher prevalence of MIAC in PPROM pregnancies. We think that differences in gestational age and ethnicity can partly explain the differences in rates of MIAC.

The strength of this study is that it provides new information concerning the amniotic fluid protein profile based on the levels of 18 mediators in response to *Ureaplasma* spp. and other bacteria in the amniotic cavity. Another important strength is that microbial invasion of the amniotic cavity was evaluated using non-cultivation techniques for both *Ureaplasma* spp. and other bacteria. A unique aspect of this study is the precise preanalytic preparation of the amniotic fluid samples, which were collected using the same technique and examiners in a single institution. However, this study has some limitations. First, the sample size of the subgroups was relatively small. Second, we are aware the fact that our approach for revealing bacteria in the amniotic fluid is not optimal for the description of polymicrobial finding. The rate of polymicrobial finding identified in our study is lower than published previously [31], [32]. However, the unraveling of amniotic fluid microbiome of pregnancies complicated by PPROM is beyond the scope of this study. Third, molecular methods can reveal the presence of bacterial DNA in the amniotic fluid regardless of its abundance; we did not quantify the amounts of bacteria in the amniotic fluid because the using of non-specific PCR approach. Fourth, we did not take into consideration the presence of viruses in the amniotic fluid.

In conclusion, *Ureaplasma* spp. elicit a protein profile of intraamniotic inflammatory response (determined by the amniotic fluid levels of 18 cytokines and mediators) that is slightly different from that of other bacteria. Nevertheless, the intensity of inflammatory response between *Ureaplasma* spp. and other bacteria seems to be very similar, when bacteria are detected using non-cultivation techniques.
